# Global trends and performance of dry needling from 2004 to 2024: a bibliometric analysis

**DOI:** 10.3389/fneur.2024.1465983

**Published:** 2024-10-09

**Authors:** Min Wang, Tianci Zhao, Jiaxin Liu, Shouyang Luo

**Affiliations:** ^1^Yunnan Baiyao Group Chinese Medicinal Resources Division, Kunming, China; ^2^First Clinical Medical College, Hubei University of Chinese Medicine, Wuhan, China; ^3^Department of Tuina and Rehabilitation Medicine, Hubei Provincial Hospital of Traditional Chinese Medicine, Wuhan, China; ^4^Department of Tuina and Rehabilitation Medicine, Affiliated Hospital of Hubei University of Chinese Medicine, Wuhan, China; ^5^Department of Tuina and Rehabilitation Medicine, Hubei Institute of Traditional Chinese Medicine, Wuhan, China; ^6^Department of Acupuncture, Huangpi District Hospital of Traditional Chinese Medicine, Wuhan, Hubei, China

**Keywords:** bibliometric analysis, dry needling, global trends, 2004, 2024

## Abstract

**Background:**

Many doctors are incorporating dry needling into their clinical practice. Despite this growing trend, there has not been a comprehensive bibliometric analysis conducted in this field. Thus, this study aims to investigate the current research landscape, key research contributors, and popular research topics in dry needling, and to analyze the developmental trends within this area of study.

**Methods:**

The study utilized the Web of Science Core Collection (WoSCC) as the main data source. Scientific literature was gathered through title (TI) searches for original clinical research papers on dry needling published between 2004 and 2024, using ‘dry needling’ or ‘dry needle’ as the search term. Statistical analyses and visualizations of the literature information, such as keywords, countries, research institutions, and authors, were conducted using the bibliometric.com online platform and VOSviewer. This approach aimed to statistically analyze and visualize the key research entities, hotspots, and frontiers in dry needling research. Additionally, the study delved into collaborative networks, research outputs, hot topics, and trends within the field of dry needling.

**Results:**

This investigation encompassed 468 publications, with the year 2021 topping the charts for the highest publication output, amassing a total of 271 articles. The journal “Acupuncture in Medicine” emerged as the most frequently cited publication. The most impactful article was titled “Acupuncture and dry-needling for low back pain: An updated systematic review within the framework of the Cochrane Collaboration.” Spain took the lead as the most productive country in this domain, with the United States closely following. Cesar Fernández-de-las-Peñas emerged as the most prolific author in the field. The Universidad Rey Juan Carlos in Spain was recognized as the most productive institution for research in dry needling. As for journal keywords, “dry needling,” “trigger point,” and “myofascial pain syn-drome” were the triumvirate of terms most recurrently encountered.

**Conclusion:**

The field of dry needling research has witnessed significant growth in recent years, characterized by the emergence of novel trends such as comparative studies with acupuncture, exploration into the mechanisms of action, and a transition toward interdisciplinary approaches. As medical models evolve, the focus is expanding from the exclusive treatment of muscle pain to broader applications. Despite this progress, the domain is underscored by a paucity of large-scale, multicenter clinical trials and animal studies. There exists an imperative for enhanced collaboration among academic and research institutions. A more profound exploration and comprehensive research endeavors are essential to enhance our understanding and broaden the clinical application of dry needling techniques.

## 1 Introduction

Dry needling, also referred to as Western acupuncture or myofascial trigger point dry needling, is a technique utilized by physical therapists and various healthcare professionals to address pain and movement disorders (1, 2). The term ‘dry needling’ was introduced to differentiate it from ‘wet needling,’ which involves the injection of fluids into the subcutaneous tissue using a syringe. Unlike wet needling, dry needling does not involve the injection of any substance; rather, it focuses on the therapeutic effects of needle insertion alone. The technique of dry needling can be broadly classified into superficial needling or deep needling, depending on the depth of needle penetration. Furthermore, it can be subdivided into trigger point dry needling, fascial needling, scar tissue needling, and other variations (3). The historical background of dry needling is closely intertwined with the quest for effective treatments for painful musculoskeletal conditions. Research exploring the origins and relief of pain through muscle tissue injections laid the foundation for the trigger point theory, which subsequently paved the way for the emergence of dry needling as a viable treatment modality.

Significant clinical findings dating back to 1941 and 1947 demonstrated that simple dry needling could provide long-lasting relief from musculoskeletal pain (4, 5). However, these findings did not garner widespread attention from the academic or clinical communities until the 1970s and 1980s, when the focus shifted to acupuncture and the scientific explanation of myofascial trigger points. Since the year 2000, there has been a notable increase in academic interest in dry needling, leading to its adoption in complementary health professions such as physical therapy, osteopathy, and chiropractic. Related studies suggest that compared to other treatments, dry needling has advantages in terms of mechanism, safety, and patient compliance (6, 7). This relates to dry needling therapy itself being an invasive procedure, which may inherently elicit strong psychological and physiological responses and might more readily lead patients to develop expectations, thereby magnifying non-specific effects (8, 9). Additionally, dry needling serves as a viable alternative to opioid analgesics and non-steroidal anti-inflammatory drugs (NSAIDs). While opioids are the reference analgesics for pain management, they are also subject to criticism. These medications may cause potentially severe and fatal adverse reactions. The ongoing opioid crisis in North America, with a rising number of deaths due to opioid overdose, serves as a tragic testament to this issue (10). There is evidence suggesting that dry needling is also a beneficial treatment for patients with scar pain and other scar-related symptoms, plantar fasciitis (11), and upper limb muscle strength hyperfunction after stroke (12).

Blinding is crucial in clinical trials as it can separate the specific intervention effect from bias by balancing all factors except the proposed mechanism of action across groups. Blinding in dry needling trials, especially participant and therapist blinding, poses a practical challenge. Inadequate blinding may lead to exaggerated intervention effects in dry needling trials. This hinders high-quality experiments to further confirm the effectiveness of dry needling and also impedes its further development (13).

This study aims to analyze global trends and performance of dry needling from 2004 to 2024 through bibliometric analysis. Bibliometric analysis is a research method that utilizes statistical and mathematical techniques to examine scientific publications and literature, offering insights into growth, impact, and knowledge structure within a particular field or topic (14). By using bibliometrics, we can pinpoint trends and shifts in dry needling research, facilitating a comprehensive understanding of the subject.

## 2 Materials and methods

The preference for WoS over Scopus was based on its reputation for superior data quality, making it the more favorable choice for conducting comprehensive bibliometric searches in the medical research community (15, 16). Studies were collected by searching for research papers published between 2004 and 2024 in the Web of Science core database, using “dry needling” or “dry needle” as search terms in the titles. To reduce bias caused by daily updates to the database, literature searches and data downloads were completed on 1 July 2024. The study included only English-language literature, while other types of papers (meeting abstracts, editorial materials, letters, and corrections) and irrelevant literature were excluded. The research strategy was formulated based on a bibliometric study (17).

## 3 Results

### 3.1 Analysis of papers

**Figure 1:**
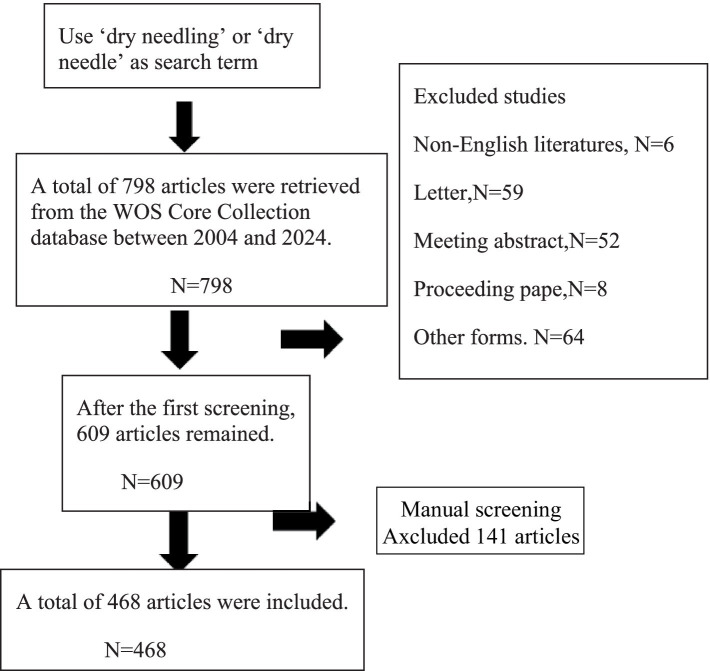
Flowchart depicting the article selection process.

Flowchart depicting the article selection process (see Figure 1).

### 3.2 Analysis of journals

**Figure 2:**
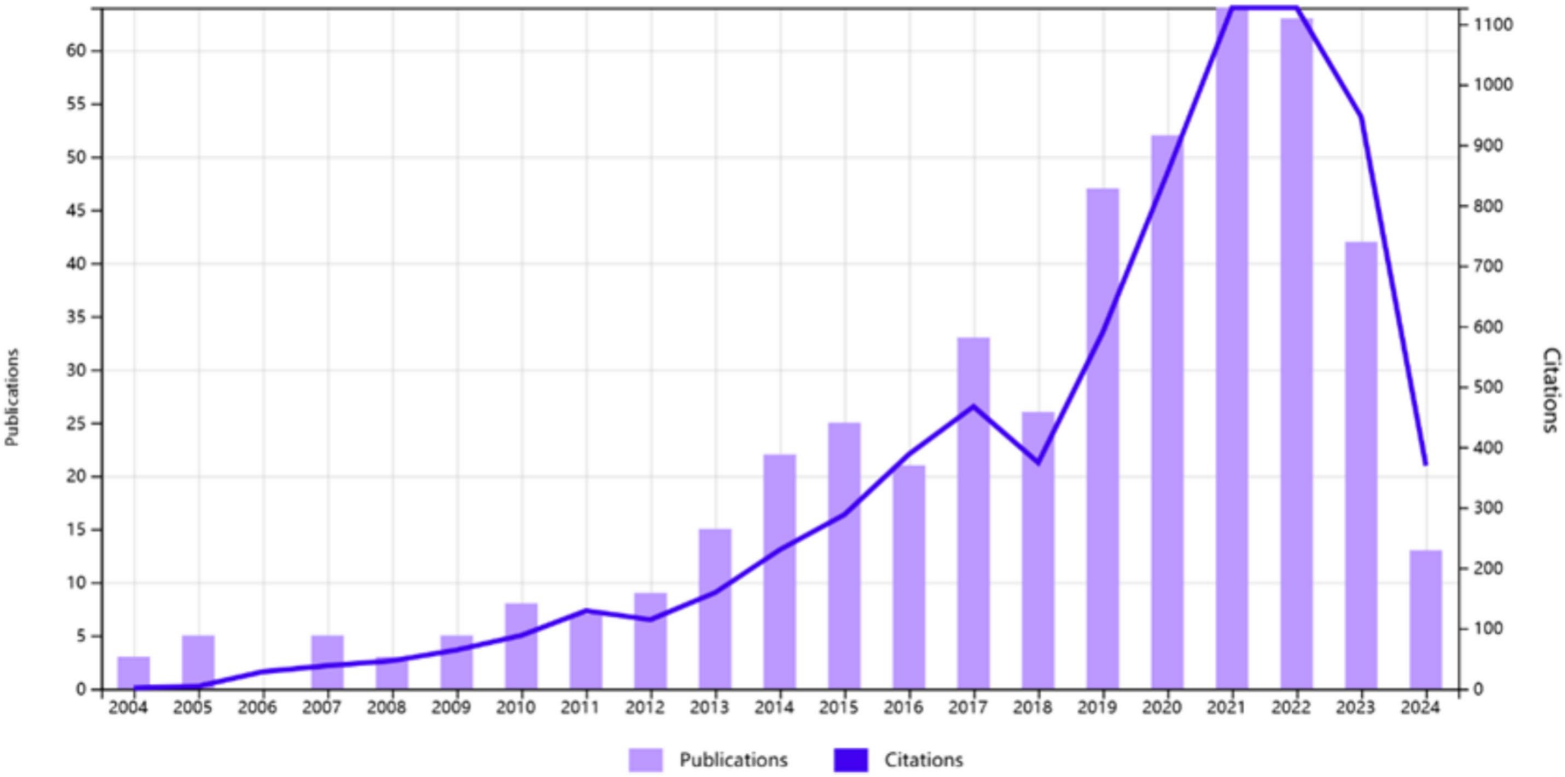
Annual trends in publication volume over the years.

The trajectory of publication volume and citation frequency in the domain of dry needling research has exhibited fluctuations across different epochs, which can be delineated into three discernible phases. The inaugural phase, spanning from 2004 to 2012, is defined by a period of slow yet steady growth, signifying the nascent stage of dry needling investigation, where the number of publications was relatively modest but trended consistently upward. The subsequent phase, from 2013 to 2018, represents a period of stability, characterized by a notable rise in publication output compared to the preceding era, with researchers sustaining a relatively consistent annual contribution to the field. The third and most recent phase, covering 2019 to 2024, is marked by an era of rapid development, where there is a pronounced surge in the number of research publications in dry needling, heralding its ascent as a burgeoning focus within the scientific community (18). Although the number of dry needling studies has decreased since 2022, the quality and depth of research appear to be continuously developing. Research focuses include comparisons between dry needling and other treatment methods, the therapeutic effects on specific diseases or symptoms, and the application of new technologies (such as ultrasound-guided dry needling therapy) (19) (Figure 2; Table 1).

**Table 1:** Citation counts and publications over time.

Rank	Journal	IF	Citations
1	Acupuncture and dry-needling for low back pain: An updated systematic review within the framework of the Cochrane Collaboration (28)	11.5	230
2	Comparison of lidocaine injection, botulinum toxin injection, and dry needling to trigger points in myofascial pain syndrome (29)	10.2	204
3	Comparison of the therapeutic effects of ultrasound-guided platelet-rich plasma injection and dry needling in rotator cuff disease: a randomized controlled trial (30)	15.17	182
4	Dry needling to a key myofascial trigger point may reduce the irritability of satellite MTrPs (31)	8.83	159
5	Ultrasound-guided dry needling and autologous blood injection for patellar tendinosis (32)	7.72	139
6	The effect of dry needling in the treatment of myofascial pain syndrome: a randomized double-blinded placebo-controlled trial (33)	10.67	128
7	Short-Term Changes in Neck Pain, Widespread Pressure Pain Sensitivity, and Cervical Range of Motion After the Application of Trigger Point Dry Needling in Patients with Acute Mechanical Neck Pain: A Randomized Clinical Trial (34)	11	121
8	Remote Effects of Dry Needling on the Irritability of the Myofascial Trigger Point in the Upper Trapezius Muscle (35)	7.4	111
9	Comparison of Laser, Dry Needling, and Placebo Laser Treatments in Myofascial Pain Syndrome (36)	4.9	103
10	Comparison of the Short-Term Outcomes Between Trigger Point Dry Needling and Trigger Point Manual Therapy for the Management of Chronic Mechanical Neck Pain: A Randomized Clinical Trial (37)	9.18	101

### 3.3 Analysis of countries/regions

**Figure 3:**
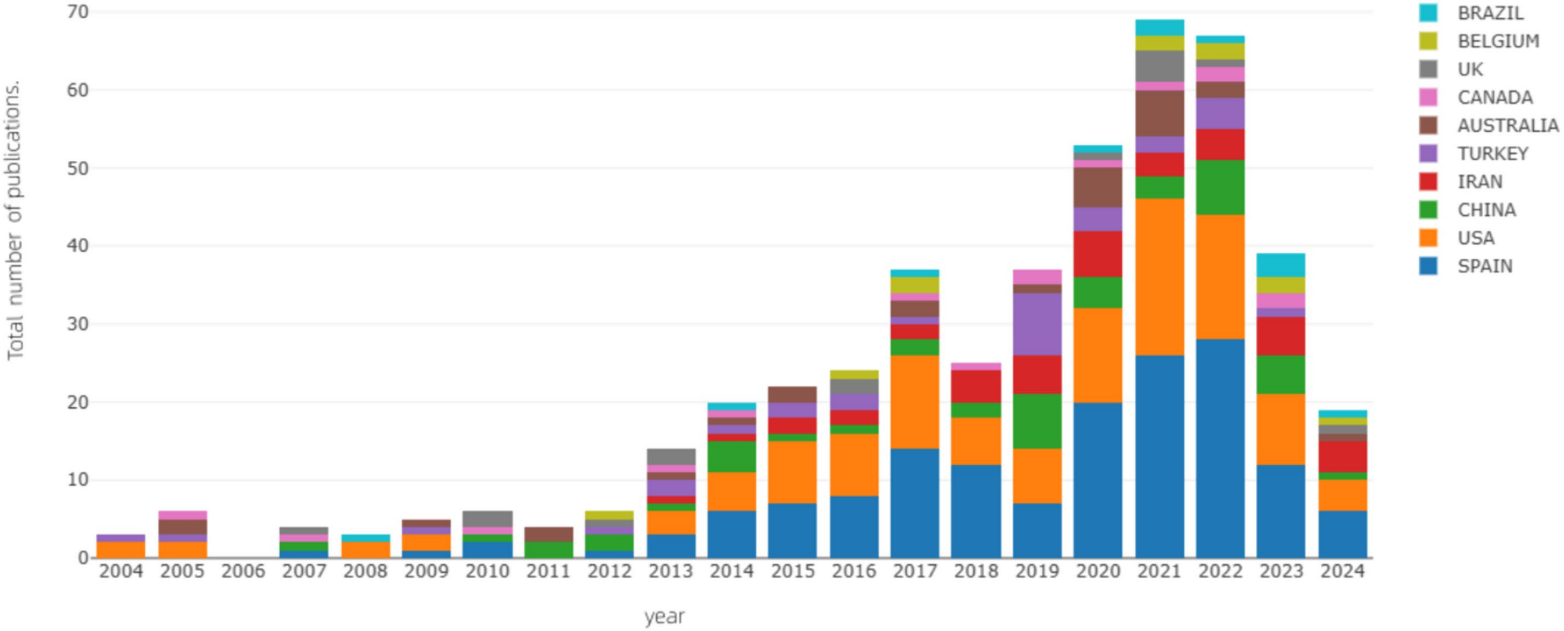
Annual publication trends by country.

The findings of this investigation reveal that the predominant countries in the domain of dry needling research are Spain (with 185 publications, constituting 39.5%), the United States (with 158 publications, comprising 33.8%), and Iran (with 45 publications, accounting for 9.6%). Notably, publications emanating from Spain and the United States far exceed those from other nations, collectively comprising 73.3% of the global publication output. Additionally, China and Iran share a similar contribution, with each nation producing 44 publications in this field (Figure 3).

### 3.4 Analysis of institutions

The color scheme of the VOSviewer network can infer the current state of collaboration among institutions; fewer colors in the VOSviewer network indicate a greater propensity for inter-institutional collaboration. From the network density, it can be deduced that there is poor cross-institutional collaboration among research institutions, with most collaborations being intra-institutional. As indicated in Table 2, considering the number of publications and centrality, the most influential institution in this field is King Juan Carlos University.

**Table 2:** Top 10 cited journals related to dry needling.

Rank	Institutions	Published articles	Cited times	First author. Times	Co-cited times
1	Univ Rey Juan Carlos	95	761	16	170
2	Univ Tehran Med Sci	31	178	13	99
3	Univ Zaragoza	29	93	4	13
4	Univ Complutense Madrid	21	95	6	8
5	Univ Alcala	20	108	8	61
6	Rey Juan Carlos Univ	17	121	2	27
7	Univ Autonoma Madrid	13	115	2	14
8	Univ San Jorge	12	53	7	28
9	George Mason Univ	12	108	3	27
10	Univ Castilla La Mancha	11	37	4	26

### 3.5 Analysis of authors

Within the realm of dry needling’s clinical application, there are presently 203 authors who have contributed to the scientific literature. The top five of these authors are enumerated in Table 3. Cesar Fernández-de-las-Peñas, affiliated with Rey Juan Carlos University, stands out as the author with the highest H-index, reaching a commendable 184. Following in the rankings are Jaime Salom-Moreno and Ricardo Ortega-Santiago, both from Spain, who are the second and third-highest-ranked authors in this field, respectively.

**Table 3:** Top five authors in the field of dry needling research ranked by H-index influence.

Rank	Author	H index	Country
1	Fernandez-de-las-Penas, Cesar	184	Spain
2	Salom-Moreno, Jaime	148	Spain
3	Ortega-Santiago, Ricardo	140	Spain
4	Hong, Chang-Zern	118	TAIWAN
5	Hsieh, Y. -L.	113	TAIWAN

### 3.6 Analysis of keywords

**Figure 4:**
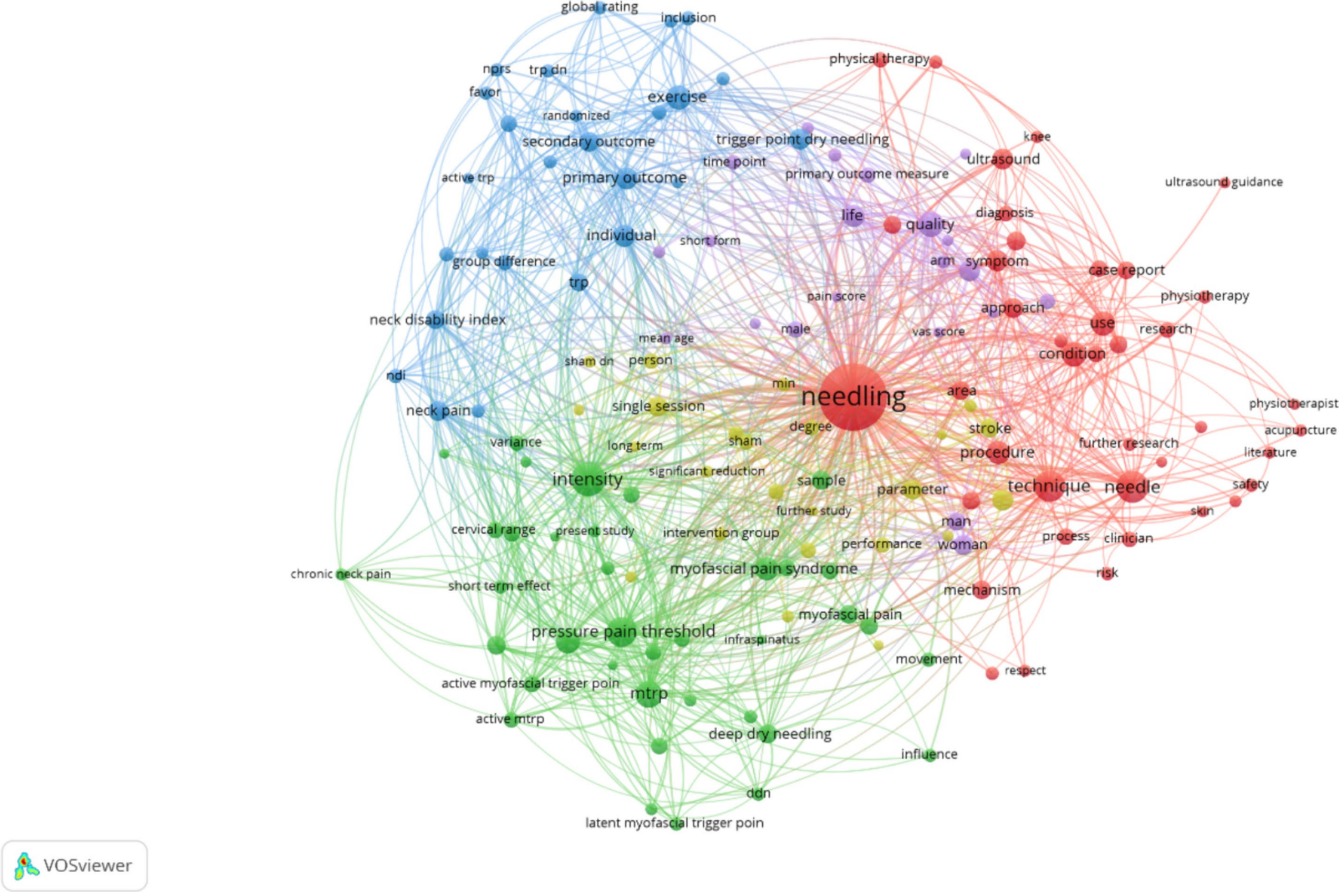
Network visualization.

The outcomes of the analysis are visually depicted as a network graph by default, as shown in Figure 4. Each node is composed of a circular shape accompanied by a label, where the dimensions of the node are governed by variables such as the degree of the node and the intensity of its interconnections. The hue assigned to each node signifies its affiliation with a particular cluster, with different colors denoting distinct clusters (20).

**Figure 5:**
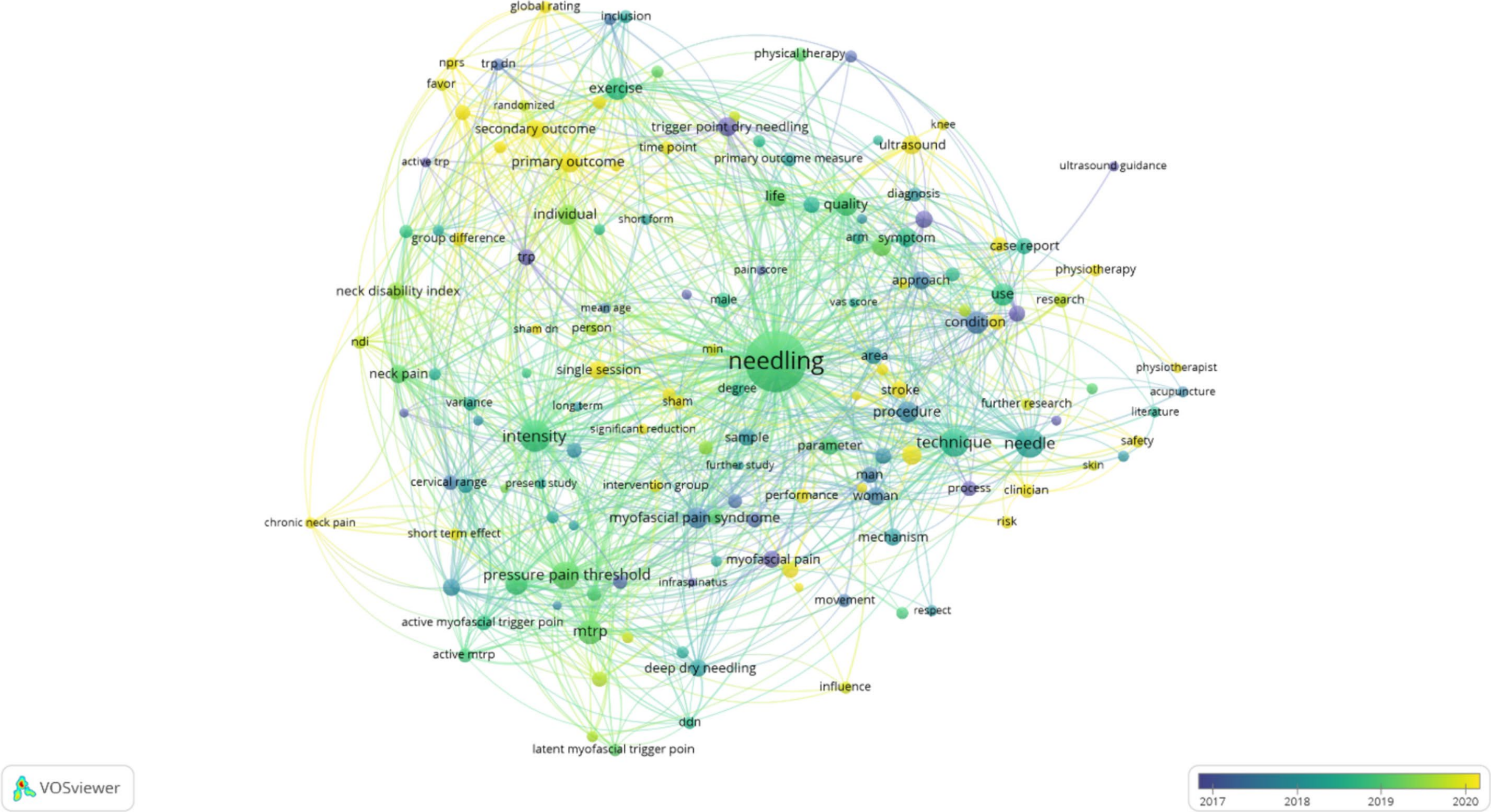
Overlay view.

The Overlay Visualization feature within the VOSviewer network constitutes a robust instrument for researchers, facilitating the extraction of valuable insights from the intricate corpus of scientific literature. This tool empowers users to conduct comprehensive analysis and derive meaningful interpretations. Unique to this feature is the capability to assign nodes with different colors, tailored to the specific research objectives of the user. As illustrated in Figure 5, the default color scheme corresponds to the average year of the keywords, providing a means to analyze the temporal progression and evolutionary trends within the field of study (21).

**Figure 6:**
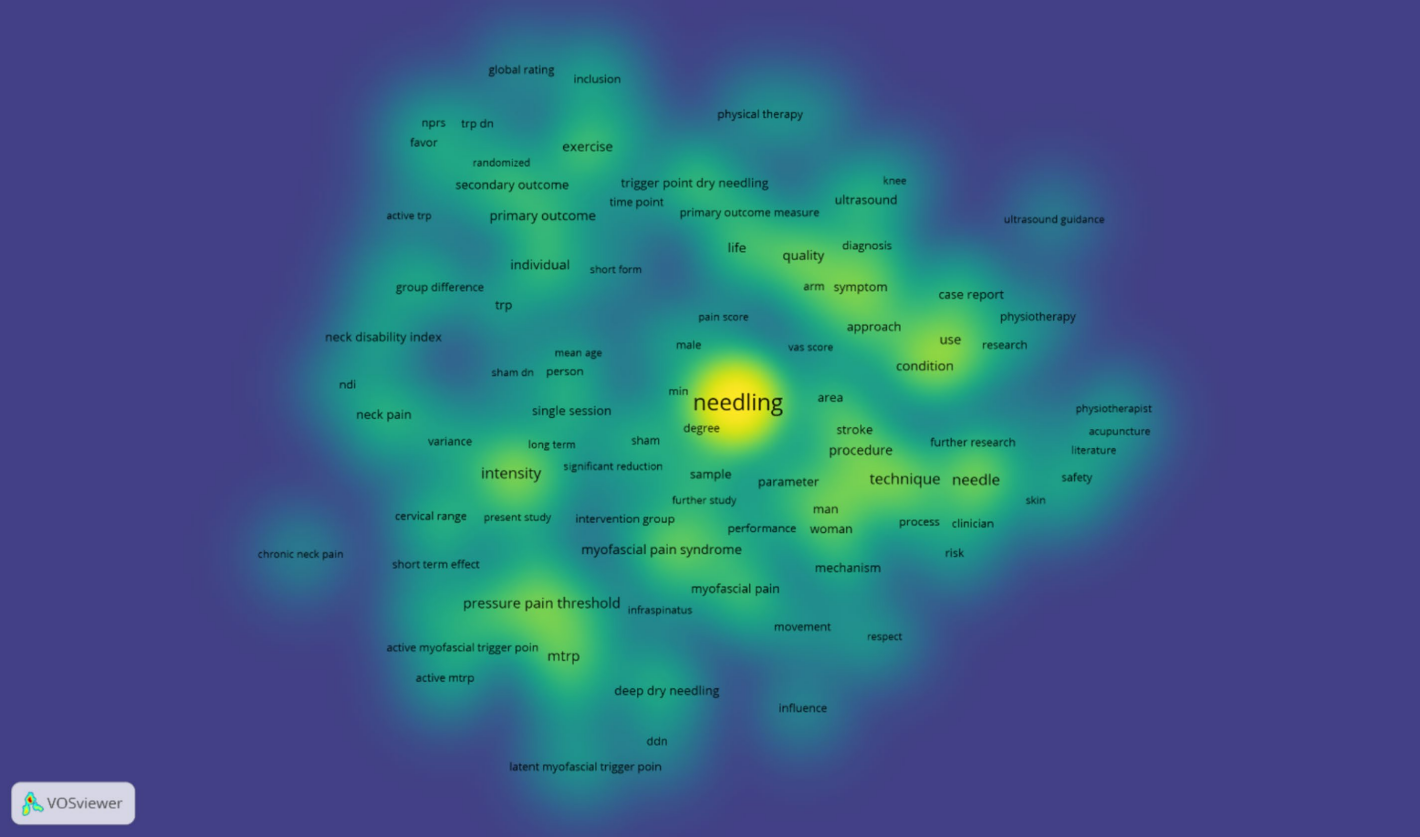
Density view.

Density visualization encompasses two modalities: item density and cluster density. Item density is denominative of the units of analysis, such as the keyword density map in the present context, wherein each unit of analysis is a keyword. Within the map, each point is assigned a color that represents the item density at that specific location (Figure 6). The default color gradient ranges from blue through green to yellow. The concentration of items in proximity to a point, along with the weight of adjacent items, determines the color intensity. A higher density and weight result in a color closer to yellow, while a lower density and weight yield a color closer to blue. The density view facilitates a rapid assessment of significant areas, and it provides insights into the distribution of knowledge and research concentration within a given field.

## 4 Discussion

The domain of dry needling research has exhibited a consistent upward trajectory in publication volume over the past two decades, with a cumulative total of 468 articles published between 2004 and 2024. Since 2008, there has been a sustained annual rise in the number of publications, signifying a burgeoning international interest and an increased focus on dry needling research. In terms of research caliber, the most cited publications include “Acupuncture and dry-needling for low back pain: An updated systematic review within the framework of the Cochrane Collaboration,” “Comparison of lidocaine injection, botulinum toxin injection, and dry needling to trigger points in myofascial pain syndrome,” and “Comparison of the therapeutic effects of ultra-sound-guided platelet-rich plasma injection and dry needling in rotator cuff disease: a randomized controlled trial,” among others. These publications are largely concentrated in the realms of rehabilitation medicine and sports medicine, and the quality of the literature has seen an improvement in lockstep with the growth in quantity and the expansion of research scope.

Research institutions are predominantly based in North America and Europe, regions characterized by higher levels of economic development and robust scientific research capabilities. A notable cluster of research institutions has emerged around Rey Juan Carlos University, with medical schools taking a leading role in research endeavors. This is attributable to the mature healthcare systems in these regions and the higher prevalence of alternative therapy usage. The Asia-Pacific region is poised for substantial growth due to the increasing incidence of musculoskeletal disorders, escalating healthcare expenditures, and the presence of skilled healthcare professionals. Overall, the dry needling research organizations are large in scale, but their interactions are weak, with a lack of collaborative research projects between institutions, resulting in relatively isolated research activities. Strengthening cooperation between institutions and authors, and fostering multicenter research projects, are pivotal strategies to enhance the quality of dry needling research. Furthermore, interdisciplinary collaboration between medical and sports research institutions, and the establishment of a robust and extensive network of connections, are essential for elevating the quality of dry needling research.

In terms of research domains, there has been a considerable number of studies not only in traditional rehabilitation fields but also in rheumatology, endocrinology and metabolism, and physiology. Research methodologies have evolved from initial clinical case reports to an increasing number of double-blind randomized controlled trials, and evaluation methods have progressed from initial assessment scales to the utilization of more sophisticated scientific research equipment. Future research in dry needling therapy is anticipated to concentrate on in-depth studies of clinical applications and the integration of dry needling with other medical specialties (22–24).

While the current study provides valuable insights into the global trends and performance of dry needling research, it is crucial to consider its broader implications for clinical practice and the potential barriers to its integration into existing pain management protocols (25). The current quality of dry needling training courses is uneven, lacking unified training standards and certification systems. This leads to varying technical levels among practitioners, affecting treatment effectiveness and safety. Establishing unified training standards and certification systems for dry needling, along with enhanced industry regulation, can ensure the safety and efficacy of dry needling. Additionally, the limited insurance coverage for dry needling therapy may hinder patients’ access to treatment and clinicians’ reimbursement. Educating insurance providers about the potential benefits and cost-effectiveness of dry needling therapy could help improve coverage options (26). In the current research landscape, there is still a lack of high-quality experiments. To validate the efficacy of dry needling, future studies should, in accordance with international recommendations, use placebo controls to enhance the reliability and replicability of the research findings (27).

## 5 Limitations

In this study, we selected the “Web of Science Core Collection” as the sole database for analysis, excluding other large medical databases. Furthermore, our research chose to filter articles based on titles. These limitations may result in the underrepresentation of potential studies in the field, thereby diminishing the impact of the research. Finally, due to the continuous updating of the database, some recently published influential papers may not have been included in our study. However, the volume of data we collected is sufficiently large to reflect the current research status in the field of dry needling to some extent.

## 6 Conclusion

Over the past 20 years, research related to dry needling has undergone rapid development, especially since 2013. This period has seen a significant increase in academic achievements in this field, which have been widely disseminated and cited. Spain and the United States are the countries with the most contributions in this area, while Spain and Taiwan are more prominent in terms of academic influence. Rey Juan Carlos University in Spain has made the most significant contributions to research in this field. The most cited article is “Acupuncture and dry-needling for low back pain: An updated systematic review within the framework of the Cochrane Collaboration,” with a citation rate of up to 230 times. Cesar Fernandez-de-las-Penas from Spain is the most prolific and highly cited author in this field. Keywords such as needling, pressure pain threshold, intensity, neck pain, chronic neck pain, physical therapy, needle technique, randomized controlled trial, and quality of life reflect the current research hotspots and future trends in this field.

## Data Availability

The original contributions presented in the study are included in the article/supplementary material, further inquiries can be directed to the corresponding authors.

## References

[1] Cagnie B Dewitte V Barbe T Timmermans F Delrue N Meeus M. Physiologic effects of dry needling. Curr Pain Headache Rep. (2013) 17:348. doi: 10.1007/s11916-013-0348-5, PMID: 23801002

[2] Legge D. A history of dry needling. J Musculo Pain. (2014) 22:301–7. doi: 10.3109/10582452.2014.883041

[3] Das S Kulkarni P. Updated review on overview of dry needling. Int J Health Sci. (2022) 5127–40. doi: 10.53730/ijhs.v6ns1.5991

[4] Budd K. Acupuncture, trigger points and musculoskeletal pain. Postgrad Med J. (1994) 70:388. doi: 10.1136/pgmj.70.823.388

[5] Lara-Palomo IC Gil-Martínez E Antequera-Soler E Castro-Sánchez AM Fernández-Sánchez M García-López H. Electrical dry needling versus conventional physiotherapy in the treatment of active and latent myofascial trigger points in patients with nonspecific chronic low back pain. Trials. (2022) 23:238. doi: 10.1186/s13063-022-06179-y, PMID: 35346331 PMC8961901

[6] Cook CE Bailliard A Bent JA Bialosky JE Carlino E Colloca L . An international consensus definition for contextual factors: Findings from a nominal group technique. Front Psychol. (2023) 14:1178560. doi: 10.3389/fpsyg.2023.1178560 37465492 PMC10351924

[7] Griswold D Learman K Rossettini G Palese A Ickert E Wilhelm M . Identifying priority gaps in contextual factors research and force-based manipulation. An international and interdisciplinary Delphi study. J Man Manip Ther. (2023) 32:118–26. doi: 10.1080/10669817.2023.2255820 37697816 PMC10795597

[8] Giacomo R Campaci F Bialosky J Huysmans E Vase L Carlino E. The biology of placebo and nocebo effects on experimental and chronic pain: state of the art. J Clin Med. (2023) 12:4113. doi: 10.3390/jcm12124113 37373806 PMC10299252

[9] Ezzatvar Y Dueñas L Balasch-Bernat M Lluch-Girbés E Rossettini G. Which portion of physiotherapy Treatments' effect is not attributable to the specific effects in people with musculoskeletal pain? A Meta-analysis of randomized placebo-controlled trials. J Orthop Sports Phys Ther. (2024) 54:391–9. doi: 10.2519/jospt.2024.12126, PMID: 38602164

[10] Busserolles J Lolignier S Kerckhove N Bertin C Authier N Eschalier A. Replacement of current opioid drugs focusing on MOR-related strategies. Pharmacol Ther. (2020) 210:107519. doi: 10.1016/j.pharmthera.2020.107519 32165137

[11] Martínez-Jiménez EM Losa-Iglesias ME Antolín-Gil MS López-López D Romero-Morales C Benito-de-Pedro M . Flexor Digitorum brevis muscle dry needling changes surface and plantar pressures: a pre-post study. Life. (2021) 11:48. doi: 10.3390/life11010048, PMID: 33451013 PMC7830844

[12] Fernández Sanchis D Cuenca Zaldívar JN Calvo S Herrero P Gómez Barrera M. Cost-effectiveness of upper extremity dry needling in the rehabilitation of patients with stroke. Acupunct Med. (2021) 40:160–8. doi: 10.1177/09645284211055750 34856821

[13] Braithwaite FA Walters JL Li LSK Moseley GL Williams MT McEvoy MP. Effectiveness and adequacy of blinding in the moderation of pain outcomes: systematic review and meta-analyses of dry needling trials. PeerJ. (2018) 6:e5318. doi: 10.7717/peerj.5318, PMID: 30083458 PMC6074757

[14] Kokol P Blažun Vošner H Završnik J. Application of bibliometrics in medicine: a historical bibliometrics analysis. Health Inf Libr J. (2020) 38:125–38. doi: 10.1111/hir.12295 31995273

[15] Kumar A. Comparing scientific productivity using Scopus and web of science (WoS): a case of Indian R&D laboratories. Asian J Technol Innov. (2020) 29:414–26. doi: 10.1080/19761597.2020.1816837

[16] Pylarinou S Kapidakis S. Tracking scholarly publishing of hospitals using MEDLINE, Scopus, WoS and Google scholar. J Hosp Librariansh. (2017) 17:209–16. doi: 10.1080/15323269.2017.1332934

[17] Ni X-J Zhong H Liu YX Lin HW Gu ZC. Current trends and hotspots in drug-resistant epilepsy research: insights from a bibliometric analysis. Front Neurol. (2022) 13:1023832. doi: 10.3389/fneur.2022.1023832 36408494 PMC9669477

[18] Huang T. Textual research of dry needling. China J Tradition Chin Med Pharm. (2017) 32:4993–6.

[19] Bureau NJ Tétreault P Grondin P Freire V Desmeules F Cloutier G . Treatment of chronic lateral epicondylosis: a randomized trial comparing the efficacy of ultrasound-guided tendon dry needling and open-release surgery. Eur Radiol. (2022) 32:7612–22. doi: 10.1007/s00330-022-08794-4, PMID: 35482125

[20] Haoteng T Guo L Fu X Wang Y Mackin S Ajilore O . Signed graph representation learning for functional-to-structural brain network mapping. Med Image Anal. (2023) 83:102674. doi: 10.1016/j.media.2022.102674 36442294 PMC9904311

[21] Yunpeng W Kan X Liu Y Ju J Yao X. Nacre-inspired layered composite gels with broad tunable mechanical strength and structural color for stress visualization. Nanoscale. (2023) 15:9060–8. doi: 10.1039/d3nr01362f 37158095

[22] Braithwaite FA Walters JL Moseley GL Williams MT McEvoy MP. A novel blinding protocol to test participant and therapist blinding during dry needling: a randomised controlled experiment. Physiotherapy. (2021) 113:188–98. doi: 10.1016/j.physio.2021.08.007, PMID: 34579950

[23] Jenkins LC Summers SJ Nasser A Verhagen A. Dry needling perceptions and experiences: a survey of Australian physiotherapists. Musculoskelet Sci Pract. (2023) 69:102895. doi: 10.1016/j.msksp.2023.102895 38081107

[24] Muñoz M Dommerholt J Pérez-Palomares S Herrero P Calvo S. Dry needling and antithrombotic drugs. Pain Res Manag. (2022) 2022:1–10. doi: 10.1155/2022/1363477, PMID: 35035647 PMC8759918

[25] Rajput K Vadivelu N. Acute pain Management of Chronic Pain Patients in ambulatory surgery centers. Curr Pain Headache Rep. (2021) 25:1. doi: 10.1007/s11916-020-00922-3, PMID: 33443656

[26] McAphee D Bagwell M Falsone S. Dry needling: a clinical commentary. Int J Sports Phys Ther. (2022) 17:551. doi: 10.26603/001c.35693 35693854 PMC9159711

[27] Rossettini G Testa M. Manual therapy RCTs: should we control placebo in placebo control? Eur J Phys Rehabil Med. (2017) 54:500–1. doi: 10.23736/S1973-9087.17.05024-9 29144109

[28] Furlan AD van Tulder M Cherkin D Tsukayama H Lao L Koes B . Acupuncture and dry-needling for low back pain: an updated systematic review within the framework of the Cochrane collaboration. Spine. (2005) 30:944–63. doi: 10.1097/01.brs.0000158941.21571.01, PMID: 15834340

[29] Kamanli A Kaya A Ardicoglu O Ozgocmen S Zengin FO Bayık Y. Comparison of lidocaine injection, botulinum toxin injection, and dry needling to trigger points in myofascial pain syndrome. Rheumatol Int. (2005) 25:604–11. doi: 10.1007/s00296-004-0485-6 15372199

[30] Dong-Wook R Park GY Kim YK Kim MT Lee SC. Comparison of the therapeutic effects of ultrasound-guided platelet-rich plasma injection and dry needling in rotator cuff disease: a randomized controlled trial. Clin Rehabil. (2012) 27:113–22. doi: 10.1177/0269215512448388 23035005

[31] Yueh Ling H . Dry needling to a key myofascial trigger point may reduce the irritability of satellite MTrPs. Am J Phys Med Rehabil. (2007) 86:397–403. doi: 10.1097/PHM.0b013e31804a554d 17449984

[32] Steven J Ali K Pocock C Robertson C Walter J Bell J . Ultrasound guided dry needling and autologous blood injection for patellar tendinosis. Br J Sports Med. (2007) 41:518–21. doi: 10.1136/bjsm.2006.034686 17387140 PMC2465422

[33] Levent T Akarsu S Durmuş O Cakar E Dinçer U Kıralp MZ. The effect of dry needling in the treatment of myofascial pain syndrome: a randomized double-blinded placebo-controlled trial. Clin Rheumatol. (2012) 32:309–15. doi: 10.1007/s10067-012-2112-3 23138883

[34] María JM-V Salom-Moreno J Ortega-Santiago R Truyols-Domínguez S Fernández-de-Las-Peñas C. Short-term changes in neck pain, widespread pressure pain sensitivity, and cervical range of motion after the application of trigger point dry needling in patients with acute mechanical neck pain: a randomized clinical trial. J Orthop Sports Phys Ther. (2014) 44:252–60. doi: 10.2519/jospt.2014.5108 24568260

[35] Tsai C-T Hsieh LF Kuan TS Kao MJ Chou LW Hong CZ. Remote effects of dry needling on the irritability of the myofascial trigger point in the upper trapezius muscle. Am J Phys Med Rehabil. (2010) 89:133–40. doi: 10.1097/PHM.0b013e3181a5b1bc, PMID: 19404189

[36] Ilbuldu E Cakmak A Disci R Aydin R. Comparison of laser, dry needling, and placebo laser treatments in myofascial pain syndrome. Photomed Laser Surg. (2004) 22:306–11. doi: 10.1089/pho.2004.22.306, PMID: 15345173

[37] Rocío L-R . Comparison of the short-term outcomes between trigger point dry needling and trigger point manual therapy for the Management of Chronic Mechanical Neck Pain: a randomized clinical trial. J Orthop Sports Phys Ther. (2014) 44:852–61. doi: 10.2519/jospt.2014.5229 25269764

